# Effects of *Rosmarinus officinalis* L. Extract on Neurobehavioral and Neurobiological Changes in Male Rats with Pentylenetetrazol-Induced Epilepsy

**DOI:** 10.3390/toxics11100826

**Published:** 2023-09-30

**Authors:** Jawaher Alrashdi, Gadah Albasher, Mohammed M. Alanazi, Wedad Saeed Al-Qahtani, Abdulkareem A. Alanezi, Fawaz Alasmari

**Affiliations:** 1 Department of Zoology, College of Science, King Saud University, Riyadh 11451, Saudi Arabia; 2 Department of Pharmacology and Toxicology, College of Pharmacy, King Saud University, Riyadh 11451, Saudi Arabiaffalasmari@ksu.edu.sa (F.A.); 3 Department of Forensic Sciences, College of Criminal Justice, Naif Arab University for Security Sciences, Riyadh 11452, Saudi Arabia; 4 Department of Pharmaceutics, College of Pharmacy, University of Hafr Al Batin, Hafr Al Batin 31991, Saudi Arabia

**Keywords:** epilepsy, pentylenetetrazol, *Rosmarinus officinalis* L., neurobehavioral changes, neurobiological changes

## Abstract

This study investigated the effect of *Rosmarinus officinalis* L. (RO) extract on neurobehavioral and neurobiological changes in male rats with pentylenetetrazol (PTZ)-induced epilepsy. Rats were assigned into five groups: (1) control rats, (2) RO-treated rats, (3) PTZ-treated rats, (4) PTZ + RO-treated rats, and (5) PTZ + valproic acid (VA)-treated rats. The PTZ-treated rats required a significantly longer time and distance to find the platform in the Morris water maze test than the control and RO-treated rats. Additionally, PTZ-treated rats showed a decrease in tendency to cross over the platform compared to PTZ group. PTZ + RO-treated rats showed decreased swimming time and distance to find the platform compared to PTZ group. PTZ + RO-treated rats showed a significant decrease in seizure score, a reduced number of myoclonic jerks, and an increased onset of the first myoclonic jerk compared to PTZ group. PTZ reduced the time required to enter the dark room in the passive avoidance learning test, which was reversed by RO treatment. Biochemical results revealed that PTZ-treated rats had higher levels of oxidative stress markers. RO significantly increased the antioxidant markers levels and maintained normal rat brain histology. This study revealed that RO can shield the brain and neural tissues from PTZ.

## 1. Introduction

According to the World Health Organization (WHO), herbal medicine delivers primary healthcare to >80% of the population in low-income countries and 60% of the global population. Phytochemicals and their chemical analogs have given rise to many clinically useful drugs for treating acute and chronic diseases [1], and additional research is being conducted on therapeutic substances from medicinal plants.

*Rosmarinus officinalis* L. (RO) is a member of the Lamiaceae family [2]. RO has been widely used in traditional medicine and cooking, especially to change and enhance flavors. It is also a highly regarded medicinal plant for cold, rheumatism, and muscle and joint pain [3]. This plant exhibits many pharmacological properties, such as antibacterial, antidiabetic, anti-inflammatory, anticancer, and antioxidant effects [4]. RO is a medicinal plant and a highly abundant source of many bioactive substances found naturally in RO’s extracts, including carnozole, carnosic acid, and triterpenes, which are also important components of nutritional supplements, complementary and alternative medicine, and antioxidants [5]. Antioxidant components decrease lipid peroxidation and interact with the detrimental free radical chain reactions [6].

Neurological disorders are characterized by impairment of the central nervous system. Older adults were found to develop neurological conditions, including dementia, Alzheimer’s disease, migraine, Parkinson’s disease, stroke, and epilepsy worldwide. Several therapeutic techniques can treat the symptoms in the early stages of these conditions; however, individuals become increasingly incapacitated as time passes and with potential drug side effects [7]. Therefore, natural products are promoted globally to maximize safety.

Epilepsy is a serious, chronic neurological disorder that affects individuals of all ages. It is strongly associated with significant morbidity, mortality, and reduced quality of life [8]. It is one of the most prevalent chronic neurological disorders worldwide and is characterized by recurrent, unpredictable, and typically unprovoked seizures. According to the WHO, at least 50 million people worldwide are affected by epilepsy, and approximately 0.5–1% of people worldwide have epilepsy [8]. Several studies have revealed bidirectional relationships between epilepsy and depression, suicidal ideation, and attention deficit disorders. Two population-based studies revealed that patients with epilepsy were four to seven times more likely to have a depression before the onset of their condition than controls [9].

Previous studies have shown the effectiveness of RO extract in induced seizures [10,11]. A study also revealed that RO acid counteracts the effects of hypoxia-ischemia and improves movement, cognition, and spatial memory [12]. RO extract has also been shown to enhance long-term memory in scopolamine-administered rats [13]. Another study revealed the neuroprotective benefits of RO in combination with fluoxetine (FLX) [14].

This study investigated the effect of *Rosmarinus officinalis* L. (RO) extract on neurobehavioral changes in male rats with pentylenetetrazol (PTZ)-induced epilepsy. RO can modulate seizure scores and memory functioning through molecular pathways. We hypothesized that RO can normalize oxidative stress and neuroinflammatory markers in rats that have developed epilepsy. Apoptotic proteins can be also modulated by RO treatments in epileptic models. 

## 2. Materials and Methods

### 2.1. Animals

Forty male Wistar rats (150–170 g, 7–9 weeks old) were provided by the Animal House of the College of Science at King Saud University, Riyadh, Saudi Arabia. During the adaptation and experimental periods, all animals were housed in plastic cages under ambient controlled conditions (25 ± 1 °C, 50 ± 10% humidity, and 12 h light/dark cycle). All study protocols were approved by the Institutional Ethics Committee (IACUC) (KSU_SE_21_13).

### 2.2. Induction of Epilepsy

This was performed as previously described [15] using repetitive intraperitoneal (i.p.) injections of PTZ (50 mg/kg) dissolved in normal saline (Cat. No. P6500, Sigma Aldric, Cambridge, UK). Accordingly, PTZ was injected every 2 days until the treated rats showed a total seizure score of 5 (development of kindling), 24 days post PTZ injection.

### 2.3. Preparation of RO Aqueous Extract

The RO leaves were bought in Riyadh, Saudi Arabia, from licensed local marketplaces. After that, distilled water was used to wash the leaves. Samples were freeze dried at −80 °C, pulverized into powder, and then frozen at −80 °C. A quantity of 500 g of powder was soaked in 1 L of methanol in a clean bottle for 24 h while being constantly stirred at about 27 °C. This phase was repeated three times. After that, the extract was run through sterile filter paper. Under low pressure, the extract had its solvent removed. After concentration, all extracts were kept at −20 °C until use [16].

### 2.4. Experimental Design

After a 1-week adaptation period, all rats were divided into five groups of eight rats each, as follows:

- Control group: Normal saline was administered.
- RO-treated group: RO extract (100 mg/kg) was administered.
- PTZ-treated group: Rats were treated with pentylenetetrazol (PTZ) (30 mg/kg).
- PTZ + RO-treated group: Rats were treated with RO (100 mg/kg) for 30 min before treatment with PTZ (30 mg/kg).
- PTZ + valproic acid (VA)-treated group (a positive control group): Rats were treated with VA (300 mg/kg) 30 min before treatment with PTZ (30 mg/kg).

All drugs were administered via oral gavage needle. The PTZ-treated rats achieved a total seizure score of 5/5 by the end of day 24; therefore, the experiments were terminated on this day.

### 2.5. Dose Selection

The dose of RO was based on a study by Naderali et al. (2018), which showed the protective potential of RO against kainic acid (KA)-induced hippocampal damage and memory deficits by suppressing neurodegeneration [17]. The dose and route of valproic acid (VA) administration were based on previous studies that showed potent anti-elliptic and neuroprotective effects in PTZ-treated rats [18]. Lower doses of 100 mg/kg also attenuated PTZ-induced catamenial epilepsy (caused by the estrous cycle) in female rats [19].

### 2.6. Scoring of Epileptic Seizures

Immediately after each PTZ treatment, each rat was placed in an individual transparent Plexiglass cage and monitored for 30 min to evaluate the seizure score using a previously established five-scale racing scoring system [15,20,21]. The following scores were considered: (A) Score 0 (no response), (B) Score 1: facial movements with saccades of ears and whiskers, (C) Score 2: myoclonic jerks without rearing, (D) Score 3: unilateral or bilateral limb clonus, (E) Score 4: rearing and forelimb clonic seizures, (F) Score 5: Generalized tonic-clonic seizures with falling.

### 2.7. Morris Water Maze (MWM) Test

After scoring the seizures, the spatial memory of all rats was tested using the MWM test as described previously [22]. The test was performed in a large swimming pool with a 1.7 m diameter and 60 cm depth. The pool was hypothetically divided into four directional quadrants (S, E, W, and N), and a hidden platform was placed in the NW quadrant 2 cm below the water surface. On the training day, all the rats were released from one quadrant and could locate and step over the visible platform. The next day (1–4), the platform was submerged in water (2 cm), and milk was added for camouflage. The test was repeated for the next 4 days (test days). The escape time (latency) to find the platform and swimming distance (m) were calculated as intelligence and intact memory indicators. An additional probe trial was conducted on day 5, when the platform was removed, and the test procedure was repeated for each rat to calculate the number of times it passed over the rescue platform.

### 2.8. Passive Avoidance Learning (PAL) Test

The emotional memory of all rats was tested using the dark room PAL test, as previously described [22]. The test apparatus contained a large illuminated room and a small dark room with an electrical grid floor. The rooms were separated by a door that could be opened or closed. The test consisted of training and test procedures. During training, each rat was placed in an illuminated area with an open door and was given three trials, each lasting 3 min, to explore the machine. In the fourth trial, the animal was allowed to re-explore, and once it entered the dark room, the door was closed, and the rat was exposed to a foot electrical stimulation (50 Hz, 1.5 mA for 2 s), after which the door was opened. All the animals were returned to their cages. Two hours later, each rat was returned to the apparatus and placed in a light room with an open door. The time required by the animals to enter the dark room was also recorded.

### 2.9. Tissue Collection and Processing

After behavioral assessment, all rats were anesthetized with a ketamine/xylazine mixture (90/10 mg/mg). Each rat’s skull was opened, and the brains were removed and placed on ice. The brains were divided into two equal parts. One portion was placed in 10% buffered formalin and processed for histological evaluation. The other halves were cut into smaller pieces and preserved at −80 ℃ for further procedures.

### 2.10. Biochemical Analysis in the Brain Homogenates

Part of the brain tissue was homogenized in phosphate-buffered saline (pH 7.4). The homogenates were centrifuged at 1000× *g* to collect the supernatants. These supernatants were stored at −20 °C and used later to measure the levels of several markers. Malondialdehyde (MDA), an indicator of lipid peroxides, was measured using an assay kit (cat no. MBS268427, MyBioSource, San Diego, CA, USA). Total levels of superoxide dismutase (SOD), tumor necrosis factor-alpha (TNF-α), glutathione peroxidase (GPX), interleukine-6 (IL-6), and glutathione (GSH) were measured using ELISA-based kits (Cat. No. RTFI00215, Cat. No. RTFI01177, Cat. No. RTEB0206, Cat. No. RTEB0061, and Cat. No. RTEB1811, all supplied by Assay Genie, London, UK). Total levels of cytochrome c, Bcl2, Bax, and caspase-3 in the homogenates were measured using ELISA (Cat. No. MBS9304546, Cat. No. MBS2881713, Cat. No. MBS935667, and Cat. No. MBS018987 MyBiosources, San Diego, CA, USA, respectively). All measurements were performed for eight samples/groups, according to the manufacturer’s instructions [23,24,25].

### 2.11. Histological Evaluations

Brain sections were preserved in 10% buffered formalin for 24 h. Tissues were rehydrated in ascending ethanol concentrations (70–100%). All the slides were cleared with xylene and embedded in paraffin. Afterward, all tissues were sectioned at 3–5 μm using a rotatory microtome. The sections were placed on glass slides and stained with hematoxylin and eosin (H&E). The mounting medium and coverslips were used to cover each section. All slides were examined under a light microscope (Nikon Eclipse E200, Tokyo, Japan) and photographed at 200× [26,27].

### 2.12. Statistical Analysis

One-way analysis of variance (ANOVA) was used to evaluate all data using GraphPad Prism software. Utilizing the Kolmogorov−Smirnov test, normality was evaluated. Tukey’s post hoc test was used to compare various groups. At *p* < 0.05, data were deemed statistically different [28].

## 3. Results

### 3.1. Scoring of Epileptic Seizures

#### 3.1.1. Assessment of Seizure Scores

**Figure 1:**
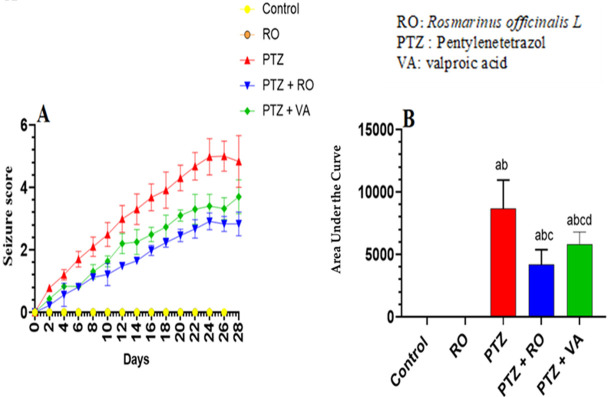
Assessment of (**A**) seizure scores during 24 days and (**B**) area under the curve of episode score in all groups of rats. Data are presented as means ± SD of 8 rats per group. Statistical significance was set at *p* < 0.05. a: significantly different when compared to control rats; b: significantly different when compared to RO-treated rats; c: significantly different when compared to PTZ-treated rats; and d: significantly different when compared to PTZ + RO-treated rats. VA, valproic acid; SD, standard deviation; RO, *Rosmarinus officinalis* L.; PTZ, pentylenetetrazol.

Kindling (seizure score 5/5) was achieved in model rats after repetitive administration of PTZ every 2 days for 24 days (Figure 1). No seizure episodes or jerks were observed in the control or RO-treated rats throughout the 24 days of the study (Figure 1A,B). The seizure score was increased in PTZ-treated rats and reached a score of 5 by the end of day 24 (Figure 1A,B). RO and VA could attenuate this effect. 

#### 3.1.2. Total Number of Myoclonic Jerks

**Figure 2:**
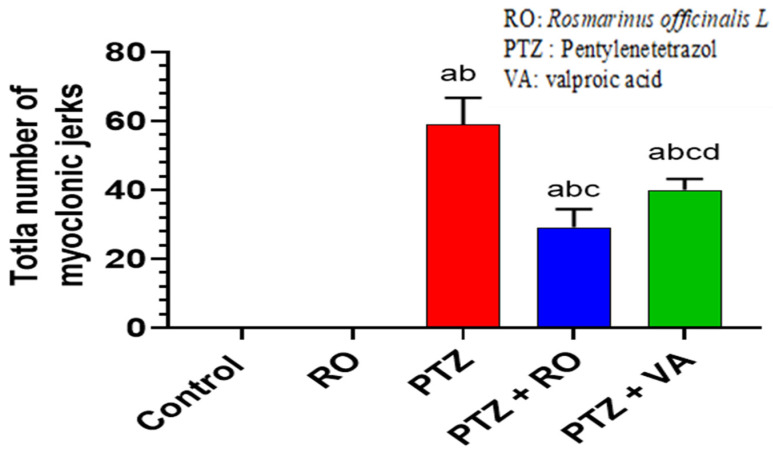
Total number of myoclonic jerks during the 24 days of all treatments in all groups of rats. Data are presented as means ± SD of 8 rats per group. Statistical significance was set at *p* < 0.05. a: significantly different when compared to control rats; b: significantly different when compared to RO-treated rats; c: significantly different when compared to PTZ-treated rats; and d: significantly different when compared to PTZ + RO-treated rats. VA, valproic acid; SD, standard deviation; RO, *Rosmarinus officinalis* L.; PTZ, pentylenetetrazol.

No myoclonic jerks were observed in the control and RO-treated rats throughout the experiment; in contrast, the number of myoclonic jerks increased significantly in the PTZ-treated rats compared to the control and RO-treated rats. The average number of myoclonic tremors was 59.7 ± 7.1 in the PTZ-treated rats. Compared with the PTZ-treated rats, the number of myoclonic jerks were decreased in the PTZ + VA-treated rats, and a more significant reduction was observed in the PTZ + RO-treated rats compared with the PTZ- and PTZ + VA-treated rats (Figure 2).

#### 3.1.3. Onset of the First Myoclonic Jerk

**Figure 3:**
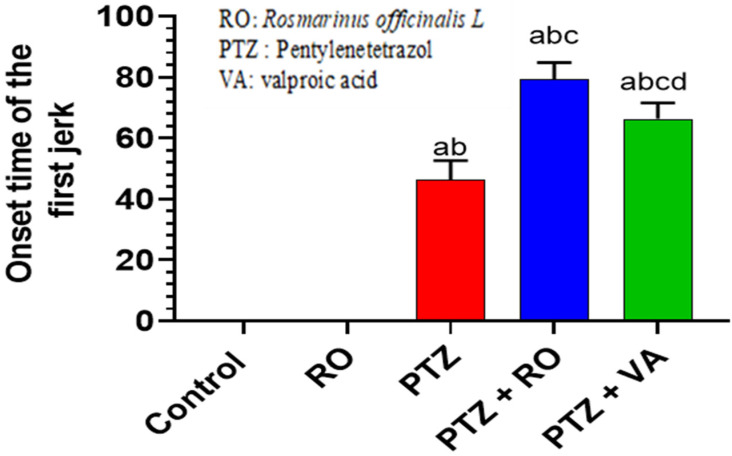
The onset of the first myoclonic jerk in all groups of rats. Data are presented as means ± SD of 8 rats per group. Statistical significance was set at *p* < 0.05. a: significantly different when compared to control rats; b: significantly different when compared to RO-treated rats; c: significantly different when compared to PTZ-treated rats; and d: significantly different when compared to PTZ + RO-treated rats. VA, valproic acid; SD, standard deviation; RO, *Rosmarinus officinalis* L.; PTZ, pentylenetetrazol.

The average onset of the first myoclonic jerk was observed after 44.8 s. Daily epilepsy shift score and AUC were reduced. The first jerk onset was significantly increased in PTZ + RO- and PTZ + VA-treated rats; however, PTZ + RO-treated rats showed a significantly longer duration of first jerk onset than PTZ + VA-treated rats. PTZ-treated rats had a lower first jerk onset time than those treated with PTZ + RO or PTZ + VA groups (Figure 3).

#### 3.1.4. Behavioral Tests

##### Time Required to Find the Hidden Platform during the MWM Test

**Figure 4:**
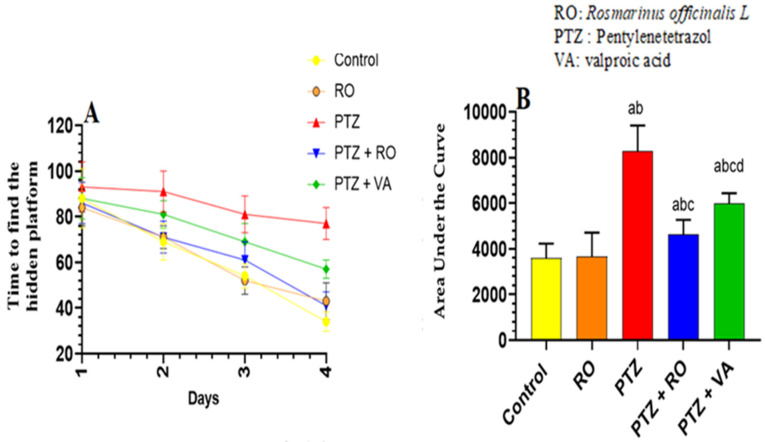
Time required to find the hidden platform (**A**) daily readings and (**B**) area under the curve in the MWM test in rats of all groups. Data are presented as means ± SD of 8 rats per group. Statistical significance was set at *p* < 0.05. a: significantly different when compared to control rats; b: significantly different when compared to RO-treated rats; c: significantly different when compared to PTZ-treated rats; and d: significantly different when compared to PTZ + RO-treated rats. VA, valproic acid; SD, standard deviation; RO, *Rosmarinus officinalis* L.; PTZ, pentylenetetrazol; MWM, Morris water maze.

A significant and progressive decline in swimming time to find the hidden platform was observed over the 4 days of the MWM test in the control and RO-treated groups. No significant variations in the time to find the hidden platform were observed between the control and RO-treated rats; however, the time to find the hidden platform, as measured on days 1–4, was significantly higher in PTZ-treated rats than in the control or RO-treated rats. Compared to rats treated with PTZ, the time to find the hidden platform was decreased in rats treated with PTZ + RO and PTZ + VA, and the time to find the hidden platform was less in PTZ + RO-treated rats than in PTZ + VA-treated rats (Figure 4A,B).

##### Total Swimming Distance to Find the Hidden Platform during the MWM Test

**Figure 5:**
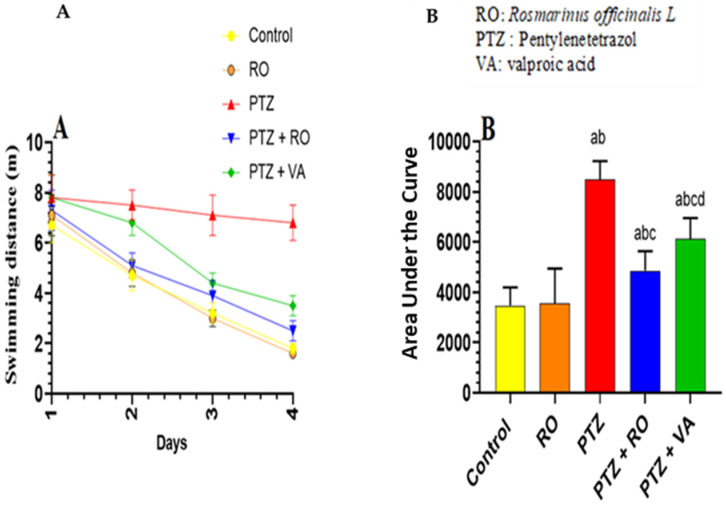
The total swimming distance to find the hidden platform (**A**) daily readings and (**B**) area under the curve in the MWM test in rats of all groups. Data are presented as means ± SD of 8 rats per group. Statistical significance was set at *p* < 0.05. a: significantly different when compared to control rats; b: significantly different when compared to RO-treated rats; c: significantly different when compared to PTZ-treated rats; and d: significantly different when compared to PTZ+RO-treated rats. VA, valproic acid; SD, standard deviation; RO, *Rosmarinus officinalis* L.; PTZ, pentylenetetrazol; MWM, Morris water maze.

Over the 4 days of the MWM test, the studied groups of rats showed significant and progressive differences in the distance required to discover the hidden platform. The distance to find the hidden platform did not differ noticeably between the RO-treated and control rats. The distance to find the hidden platform was significantly greater in the PTZ-treated rats than in the RO-treated or control rats. Compared to PTZ-treated rats, the distance to find the hidden platform decreased in the PTZ + RO- and PTZ + VA-treated rats and was reduced in the PTZ + RO-treated rats compared to PTZ + VA group (Figure 5A,B).

##### Number of Crossing Times over the Removed Platform during the Probe Trial of the MWM Test

**Figure 6:**
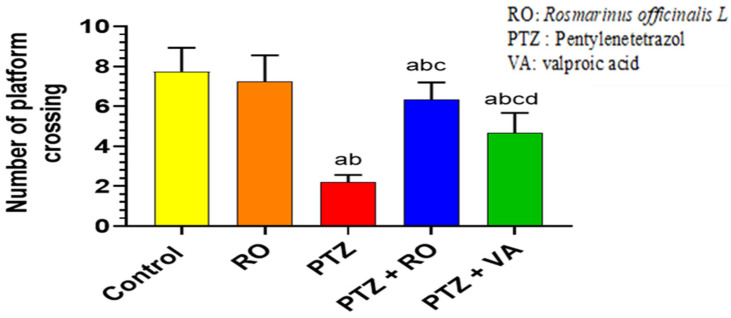
The number of crossing times over the removed platform during the probe trial of the MWM test in rats of all groups. Data are presented as means ± SD of 8 rats per group. Statistical significance was set at *p* < 0.05. a: significantly different when compared to control rats; b: significantly different when compared to RO-treated rats; c: significantly different when compared to PTZ-treated rats; and d: significantly different when compared to PTZ + RO-treated rats. VA, valproic acid; SD, standard deviation; RO, *Rosmarinus officinalis* L.; PTZ, pentylenetetrazol; MWM, Morris water maze.

There were no significant differences in the number of crossings over the removed platform in the probe trial between the control and RO-treated rats (Figure 6). Compared to the control and RO-treated rats, PTZ-treated rats significantly decreased the number of crossings over the removed platform. Compared with PTZ-treated rats, PTZ + RO- and PTZ+VA-treated rats exhibited an increase in crossings over the platform, and the number of crossings was increased higher in PTZ + RO-treated rats compared to PTZ-VA group (Figure 6).

##### PAL Test

**Figure 7:**
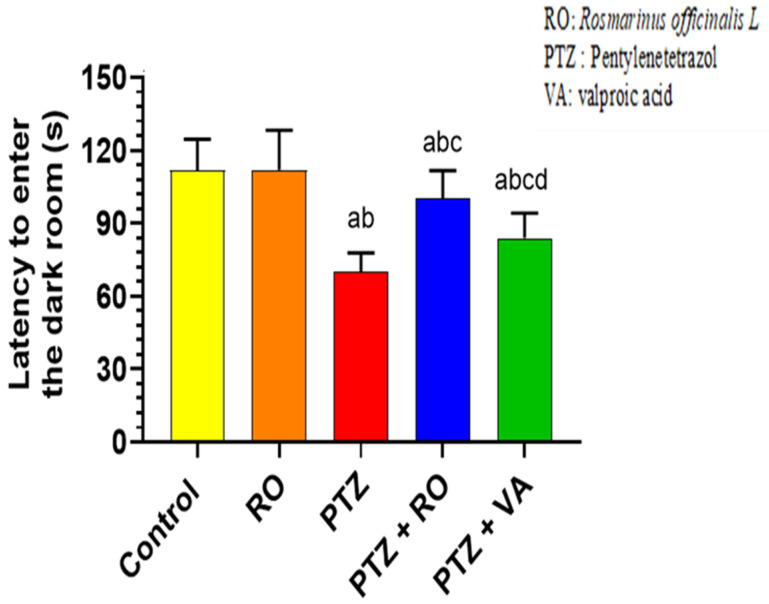
The time each rat spent entering the darkroom during the PAL test in rats of all groups. Data are presented as means ± SD of 8 rats per group. Statistical significance was set at *p* < 0.05. a: significantly different when compared to control rats; b: significantly different when compared to RO-treated rats; c: significantly different when compared to PTZ-treated rats; and d: significantly different when compared to PTZ + RO-treated rats. VA, valproic acid; SD, standard deviation; RO, *Rosmarinus officinalis* L.; PTZ, pentylenetetrazol; PAL, passive avoidance learning.

The time required to enter the dark area in the PAL test was not significantly different between the control and RO-treated rats. In addition, compared with control or RO-treated rats, PTZ-treated rats required less time to enter the dark area during the PAL test. Moreover, PTZ + RO- and PTZ + VA-treated rats showed an increased time required to enter the dark room compared to PTZ-treated rats; however, PTZ + VA-treated rats showed a decrease in time required to enter the dark room compared to PTZ + RO-treated rats (Figure 7).

#### 3.1.5. Biochemical Analysis

##### Levels of Antioxidant Markers

**Figure 8:**
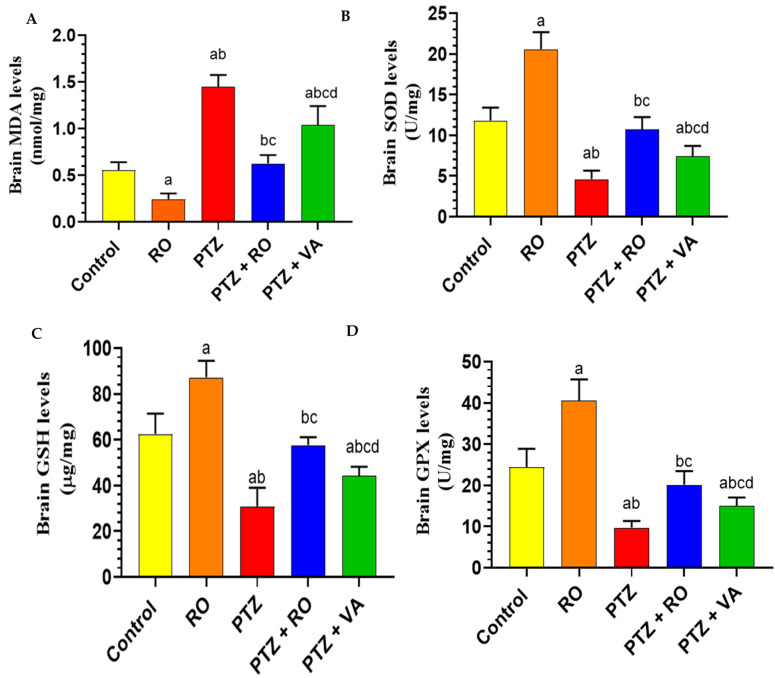
Levels of (**A**) MDA, (**B**) total SOD, (**C**) total GSH, and (**D**) total GPX in the brains of all groups of rats. Data are presented as means ± SD of 8 rats per group. Statistical significance was set at *p* < 0.05. a: significantly different when compared to control rats; b: significantly different when compared to RO-treated rats; c: significantly different when compared to PTZ-treated rats; and d: significantly different when compared to PTZ+RO-treated rats. VA, valproic acid; SD, standard deviation; RO, *Rosmarinus officinalis* L.; PTZ, pentylenetetrazol; MDA, malondialdehyde; SOD, superoxide dismutase; GSH, glutathione; GPX, glutathione peroxidase.

MDA levels were significantly reduced in the brains of RO-treated rats compared to the control rats. The brain homogenates of PTZ-treated rats showed a significant increase in the MDA levels compared to control and RO groups, and MDA levels were significantly reduced in the PTZ + RO and PTZ + VA groups compared to PTZ group. However, the MDA levels were significantly lower in PTZ + RO-treated rats than in the PTZ + VA-treated rats (Figure 8A). SOD levels were significantly increased in the brains of RO-treated rats compared to those of the control rats. Brain homogenates of PTZ-treated rats showed a significant decrease in total SOD compared to those of the control and RO-treated rats. SOD levels were significantly higher in the PTZ + RO- and PTZ + VA-treated rats than in the PTZ-treated rats. However, SOD levels was significantly higher in the PTZ + RO-treated rats than in the PTZ + VA-treated rats. Furthermore, no significant differences were observed in the levels of these markers when PTZ + RO-treated rats were compared to control rats (Figure 8B). GSH levels in RO-treated rats increased significantly compared to in the control rats; in contrast, GSH levels were decreased significantly in the PTZ-treated rats compared to the control and RO-treated rats.

Additionally, GSH levels were increased significantly in the PTZ + RO- and PTZ + VA-treated rats compared to in the PTZ-treated rats (Figure 8C). GPX levels were significantly increased in the RO-treated rats compared to in the control rats; however, there was a significant decrease in GPX levels in the PTZ-treated rats compared to the control and RO-treated rats. Additionally, there was an increase in GPX levels in the PTZ + RO- and PTZ + VA-treated rats compared to the PTZ-treated rats (Figure 8D).

##### Levels of Inflammatory Markers

**Figure 9:**
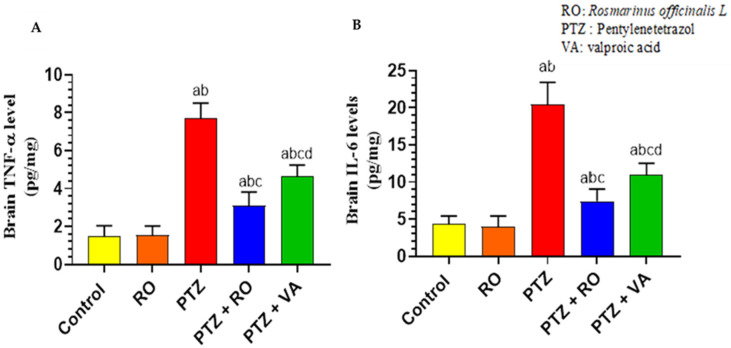
Levels of (**A**) TNF-α and (**B**) IL-6 in the brains of all groups of rats. Data are presented as means ± SD of 8 rats per group. Statistical significance was set at *p* < 0.05. a: significantly different when compared to control rats; b: significantly different when compared to RO-treated rats; c: significantly different when compared to PTZ-treated rats; and d: significantly different when compared to PTZ + RO-treated rats. VA, valproic acid; SD, standard deviation; RO, *Rosmarinus officinalis* L.; PTZ, pentylenetetrazol; TNF- α, tumor necrosis factor; IL-6, interleukin-6.

The brain homogenates of the PTZ-treated rats showed a significant increase in the levels of TNF-α as compared to the control and RO-treated rats. In contrast, the levels of TNF-α were significantly reduced in the brains of the PTZ + RO- and PTZ + VA-treated rats compared to the PTZ-treated rats. However, the brain levels of TNF-α were significantly lower in the PTZ + RO-treated rats than in the PTZ + VA-treated rats. In contrast, the levels of TNF-α remained significantly higher in the brains of the PTZ + RO and PTZ + VA -treated rats than in the control rats (Figure 9A). The RO-treated rats and the control rats did not show any significant variation in the brain levels of IL-6. When compared to the control and RO-treated rats, the brain homogenates of model rats that had received PTZ treatment revealed significantly higher levels of IL-6; in contrast, the brain IL-6 levels in the PTZ + RO- and PTZ + VA-treated rats were both lower than those in the PTZ-treated rats. Although brain IL-6 was significantly lower in the PTZ + RO-treated rats than in the PTZ + VA-treated rats, it was also significantly higher in the PTZ + RO-treated rats than in the control rats (Figure 9B).

##### Levels of Apoptotic Proteins in the Brain

**Figure 10:**
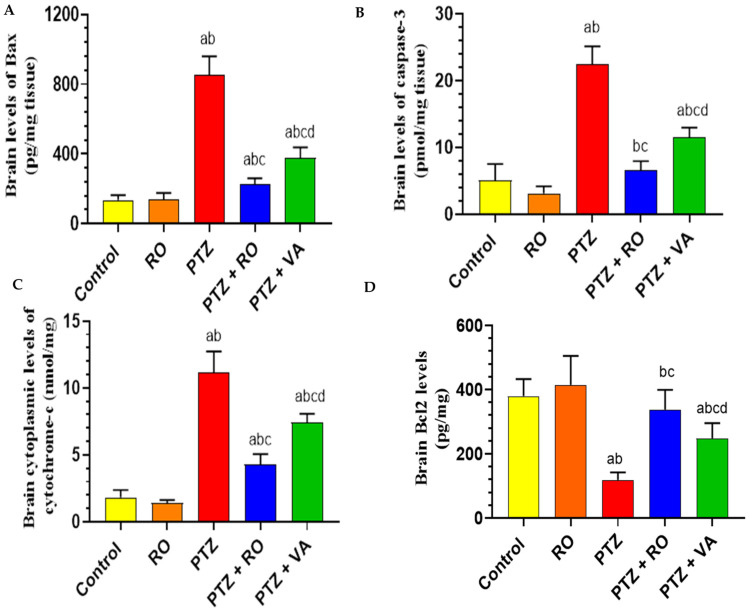
Levels of (**A**) Bax protein, (**B**) caspase-3, (**C**) cytochrome c, and (**D**) Bcl2 in the brains of all groups of rats. Data are presented as means ± SD of 8 rats per group. Statistical significance was set at *p* < 0.05. a: significantly different when compared to control rats; b: significantly different when compared to RO-treated rats; c: significantly different when compared to PTZ-treated rats; and d: significantly different when compared to PTZ + RO-treated rats. VA, valproic acid; SD, standard deviation; RO, *Rosmarinus officinalis* L.; PTZ, pentylenetetrazol.

The brain levels of Bax protein were not significantly different between the PTZ-treated, RO-treated, and control rats. Bax protein levels were significantly higher in the brains of the PTZ-treated rats than in the control and RO-treated rats (Figure 10A). Brain homogenates of the PTZ + RO- and PTZ + VA-treated rats showed a significant decrease in Bax protein levels compared with the PTZ-treated rats. Bax protein levels were significantly higher in the PTZ + VA-treated rats than in the PTZ + RO-treated rats (Figure 10A). As shown in Figure 10B, brain levels of caspase-3 were slightly lower in the RO-treated rats than in the control rats. Caspase-3 levels were significantly higher in the brains of the PTZ-treated rats than in the control and RO-treated rats (Figure 10B). Brain homogenates of the PTZ + RO- and PTZ + VA-treated rats showed a significant decrease in caspase-3 levels compared with the PTZ-treated rats. Levels of caspase-3 were higher in the PTZ + VA-treated rats than in the PTZ + RO-treated rats. Compared with the control rats, RO-treated rats had lower brain cytochrome c levels. The PTZ-treated rats had significantly higher cytochrome c levels in their brains than the RO-treated rats, and brain homogenates from the PTZ + RO- and PTZ + VA-treated rats revealed a substantial drop in cytochrome c compared to PTZ group. The PTZ + VA-treated rats had higher cytochrome c levels than the PTZ + RO-treated rats (Figure 1C). There was no statistically significant difference in the brain levels of Bcl2 between the RO-treated and control rats, and the brains of the PTZ-treated rats showed considerably reduced Bcl2 levels compared to controls (Figure 10D).

The brain homogenates of PTZ + RO- and PTZ + VA-treated rats showed a significant increase in Bcl2 levels compared to PTZ-treated rats; in contrast, Bcl2 levels were significantly lower in the brains of the PTZ + VA-treated rats than in the PTZ + RO-treated rats (Figure 10D).

#### 3.1.6. Histological Analysis of Dental Gyrus of the Hippocampi

**Figure 11:**
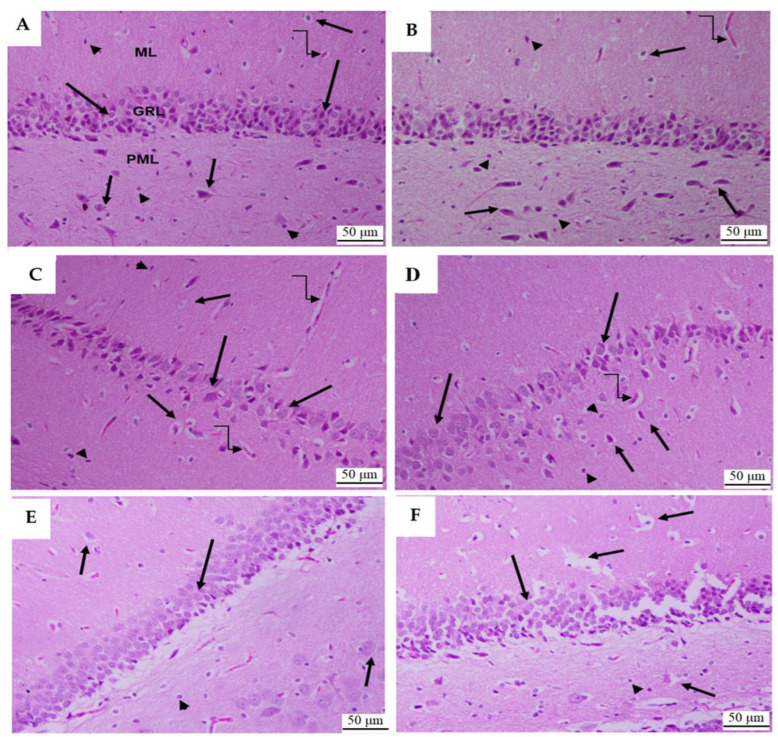
The histological section of the dental gyrus of the hippocampi of all groups of rats. (**A**) and (**B**) Taken from control rats, showing a normal structure composed of three intact layers, namely, the glandular layer (GRL) lying between the polymorphic (PML) and molecular layers (ML). The GRL comprises 4–6 cell layers with rounded pale vesicular nuclei (long arrow). The basket cells (short arrow) and glial cells (arrowhead) were also observed, and the PLM and ML layers have normally sized blood vessels (curved arrow). (**C**,**D**) Taken from PTZ-treated rats and showing an obvious reduction in the number of cell layers forming the GRL with swelling cells (long arrow). A lower number of basket and glial cells (shot arrow and arrowhead, respectively) were also observed in this group of rats with dilated blood vessels (curved arrow). (**E**,**F**) Taken from the PTZ + RO- and PTZ + VA-treated rats, respectively, and showing significant improvement in the structure of their hippocampi, with an obvious increase in the number of cell layers forming the GRL, as well as an increase in the number of basket (short arrow) and glial cells (arrowhead) of the PLM and ML. Both groups also showed normally sized blood vessels. However, some degeneration in the GRL and basket cells (short arrow) is still seen in the PTZ + VA-treated rats.

As shown in Figure 11A,B, the dental gyrus area of the hippocampi of the control and RO-treated rats showed normal features, with the presence of all three layers (glandular, polymorphic, and molecular layers) and normally sized blood vessels. The molecular layer showed 4–6 intact layers of cells, and the polymorphic and molecular layers showed many baskets and glial cells. According to Figure 11C,D, the PTZ-treated hippocampi showed an obvious reduction in the number of cell layers forming the glandular layer; in contrast, the remaining cells showed swelling. In addition, this group of rats showed reduced basket and glial cells and dilation of their blood vessels.

The hippocampi of the PTZ + RO- and PTZ + VA-treated rats showed an obvious improvement in their dental gyrus structures. They had normally sized blood vessels and an almost normal count of basket and glial cells in the molecular and polymorphic layers (Figure 11E,F). However, some degeneration in the glandular layer and basket cells was observed in the dental gyrus of the PTZ + VA-treated rats (Figure 11F).

## 4. Discussion

Herbs have a long history of being effective in treating diseases, with few side effects or toxicity [29]. Although many antiepileptic medications have been developed, most do not improve the cognitive impairment caused by refractory epilepsy. These medications also have numerous adverse effects, such as psychosis, heightened irritability, and aggressive behavior [30,31]. Different RO extraction and purification methods and numerous antioxidant assays have shown that RO is rich in phenolic compounds, such as carnosic acid and carnosol. RO oil’s antioxidant, antimicrobial, and cognitive properties and its extracts have also been investigated. Therefore, they provide a number of naturally derived antioxidants recognized by the food industry [32]. Numerous studies have highlighted the neuropharmacological benefits of RO extracts. RO has significant antibacterial, anti-inflammatory, antioxidant, anti-tumor, anti-pain, and neuroprotective effects. It also has significant clinical benefits for mood, memory, learning, pain, anxiety, and sleep [33].

In a previous study [10], treating animals with different doses of RO extract delayed the onset of picrotoxin-induced seizures, and the most effective dose was 50 mg/kg. These results are consistent with those of the present study, in which treatment of rats with RO extract (100 mg/kg) delayed the onset of seizures caused by PTZ (30 mg/kg). These results agree with Boroushaki, 2002. It was found that the most effective dose of the plant extract (12 mL/kg) reduced the onset of seizures, their duration, and the number of deaths after 24 h in PTZ-induced seizures, and the results showed that all concentrations of the plant extract delayed seizure onset [11]. In a previous study [34] consistent with our results, it was found that the latency to myoclonic jerks and generalized seizures were increased in the PTZ model treated with rosmarinic acid (30 mg/kg); moreover, the latency to myoclonic jerks induced by pilocarpine was delayed following rosmarinic acid treatment. This study also found that the appearance of spontaneous recurrent seizures was not abolished with rosmarinic acid in a chronic epilepsy model. However, rosmarinic acid could increase the time of immobility using forced swim assay, and increase the time spent at the center and crossing numbers using the open field test.

The effect of the aerial portions of RO in rats undergoing the MWM test and the effects of the essential oil on intact memory and scopolamine-induced learning were examined. Rats were administered an intraperitoneal injection of the oil 30 min before training for 5 days in a row, and the latency time to discover the platform was reduced when the oil was administered at levels of 125–250 mg/kg. Rats with learning deficits due to hyoscine (0.5 mg/kg) were also tested to determine the effect of the RO oil. The memory-impairment effects of hyoscine were reduced by RO oil [35]. This is consistent with the results of this study, which showed that RO improved learning and memory. A previous study revealed that RO significantly improved movement problems, cognition, and spatial memory [12]. In addition, these results are consistent with Rad et al., 2021, who found that RO extract (100 mg/kg) improved spatial memory retrieval using the MWM test. There was also a significant difference in the time each group spent in the target quadrant during the experiment [36].

The results of this study showed that RO extract (100 mg/kg) significantly improved long-term memory during the PAL test, which is consistent with a previous study [37] where it was found that doses (50 and 100 mg/kg) of the natural oil of RO resulted in significant improvements in long-term memory. These results also agree with Ozarowsk et al., 2013, who found that RO extracts enhanced long-term memory in rats administered scopolamine [13]. This study showed that treatment with RO extract in a rat model of epilepsy led to a decrease in lipid peroxide (MDA) levels, which agrees with the findings of recent studies [38,39,40]. Treatment with RO extract significantly increased antioxidant enzymes, such as SOD, GSH, and GPX; the results of this study agreed with prior studies [41,42]. Administration of RO increased the levels of all these enzymes and a glutathione-reducing antioxidant.

Other studies have confirmed the results of this study, which showed that RO in separate dosage forms (RO essential oil, aqueous extract, and crude plant powder) enhanced the activity of SOD and the total antioxidant capacity [43]. In a recent study, doses of RO increased the levels of several enzymes, including SOD [44]. Increased blood flow and vascular permeability, and the buildup of fluid, leukocytes, and inflammatory mediators, including cytokines, are characteristics of inflammation in its acute phase, an immediate response to tissue injury. IL-1 and TNF-α, two cytokines that are extremely strong inflammatory agents, are important in mediating acute inflammatory reactions [45]. Our work suggests that RO showed the ability to restore the neuroinflammatory cytokines levels in animal models of epilepsy. The production and expression of extracellular SOD were stimulated by pro-inflammatory cytokines [46]. Mitochondrial SOD in fibroblasts was stimulated following exposure to inflammatory cytokines such as TNF-α [46]. Here, we found that the PTZ group had higher brain antioxidant parameters, including SOD, which suggest a compensatory feedback mechanism for increased brain inflammatory cytokine levels [47]. Through the suppression of immune cell infiltration and immunological responses, extracellular SOD may produce blocking effects on IL-23-induced-inflammatory conditions such as psoriasis [48]. In response to lipopolysaccharide, SOD 3 was released from intracellular stores of macrophages producing biological responses [49]. Lactate has been considered a useful seizure-like activity biomarker, as demonstrated by an increase in its levels in the extracellular environment of the human hippocampus during seizures; rosmarinic acid (30 mg/kg/body volume) was able to reduce pilocarpine-stimulated lactate release in the brains of epileptic male rats [50]. Gap junctions that allow direct transfer of ions and molecules between neighboring cells, and gap junctions between astrocytes, play an important role in the development of brain diseases such as epilepsy [51]. Astrocytic coupling entirely disappears in hippocampal sclerosis, according to functional data from human patients and animal models, while the gap-junction-forming proteins connexin43 and connex30 are still present. Additionally, astrocyte dissociation was found to be a contributing factor in the onset of temporal lobe epilepsy [52]. In a recent study [53], human SH-SY5Y neuronal-like cells and A-172 glial-like cells were used in a hypoxic environment in vitro, examining the effects mediated by spray-dried RO extract (SDROE) on cell viability, apoptosis, and Cx43-based intercellular communication. It was discovered that SDROE had a protective effect on cells damaged by glucose deficiency (OGD), promoting metabolic turnover and cell survival while reducing Cx43-based cell coupling.

Treatment with PTZ led to decreased glial cells and expansion of blood vessels; in contrast, these effects were mitigated by treatment with RO. These results agree with those of a recent study conducted by Atef et al. (2021), where it was found that treatment with RO extract can reduce the toxic effects of monosodium glutamate (MSG) [14]. In a rat brain tissue study, the MSG group showed glial cell shrinkage and significant vasodilation, which was attenuated by RO extract and/or FLX treatment. Treatment with RO extract and FLX reduced the toxic effects of MSG in the rat hippocampus. Our results agree with those of Rad et al. (2021), who studied the beneficial effects of rosemary extract and adipose-tissue-derived stem cells on memory and hippocampal neurogenesis in Parkinson’s rat models [36]. The hippocampi of rats administered RO extract or water contained significantly more neurons than the treatment groups.

## 5. Conclusions

This study investigated the preventive and therapeutic efficacies of RO in promoting brain health in rats with epilepsy. Based on these proven properties in many studies, RO is a promising candidate for attenuating epilepsy. Therefore, RO can protect the brain via its antioxidant (GSH, SOD, and GPX), anti-apoptotic, and anti-inflammatory properties, as depicted following the oxidative stress, an effect associated with improved neurobehavioral parameter. Future studies are warranted to link the positive behavioral and neuroinflammation effects of RO to the gene and protein levels of the involved pathways and biomarkers. Future studies can investigate and compare RO treatment durations on the neurobehavioral changes in epilepsy models.

## Data Availability

The data presented in this study are available within the article.
